# Research Progress on Antibacterial Activities and Mechanisms of Natural Alkaloids: A Review

**DOI:** 10.3390/antibiotics10030318

**Published:** 2021-03-19

**Authors:** Yumei Yan, Xing Li, Chunhong Zhang, Lijuan Lv, Bing Gao, Minhui Li

**Affiliations:** 1 Department of Pharmacy, Baotou Medical College, Baotou 014040, China; yym151447@163.com (Y.Y.); lx15704967759@163.com (X.L.); zchlhh@126.com (C.Z.); 2 Inner Mongolia Key Laboratory of Characteristic Geoherbs Resources Protection and Utilization, Baotou Medical College, Baotou 014040, China; 3 Inner Mongolia Engineering Research Center of the Planting and Development of Astragalus Membranaceus of the Geoherbs, Baotou Medical College, Baotou 014040, China; 4 Department of Basic Science, Tianjin Agricultural University, Tianjin 300384, China; lv_lijuan@aliyun.com; 5 Pharmaceutical Laboratory, Inner Mongolia Institute of Traditional Chinese Medicine, Hohhot 010020, China

**Keywords:** alkaloid, antibacterial activity, drug resistance, natural product

## Abstract

Alkaloids are nitrogen-containing heterocyclic compounds typically isolated from plants. They represent one of the most important types of natural products because of their large number and structural diversity and complexity. Based on their chemical core structures, alkaloids are classified as isoquinolines, quinolines, indoles, piperidine alkaloids, etc. In-depth analyses of alkaloids have revealed their antibacterial activities. To date, due to the widespread use of antibiotics, the problem of drug-resistant bacterial infections has been gradually increasing, which severely affects the clinical efficacy of antibacterial therapies and patient safety. Therefore, significant research efforts are focused on alkaloids because they represent a potentially new type of natural antibiotic with a wide antibacterial spectrum, rare adverse reactions, and a low tendency to produce drug resistance. Their main antibacterial mechanisms include inhibition of bacterial cell wall synthesis, change in cell membrane permeability, inhibition of bacterial metabolism, and inhibition of nucleic acid and protein synthesis. This article reviews recent reports about the chemical structures and the antibacterial activities and mechanisms of alkaloids. The purpose is to solve the problem of bacterial resistance and to provide a certain theoretical basis and research ideas for the development of new antibacterial drugs.

## 1. Introduction

Bacteria are one of the general types of organisms, mainly composed of the cell membrane as the innermost layer of the cell envelope, the cytoplasm with ribosomes, and the nucleoid. Based on their general morphology, bacteria can be divided into cocci, bacilli, and spirochetes [1]. They significantly influence human activities. In particular, some common bacterial pathogens, such as *pyogenic cocci*, *Enterobacter*, and *Vibrio*, which can cause tetanus, typhoid, pneumonia, and other diseases, pose a serious public health threat [2]. The introduction of antibiotics into clinical practice has saved the lives of many patients. Penicillins, cephalosporins, new *β*-lactams, aminoglycosides, macrolides, lincomycin, and quinolones are the main types of antibiotics that treat bacterial infections by inhibiting the growth of pathogenic bacteria and killing the causative agents [3]. However, the excessive use of antibiotics has led to the emergence of drug resistance among bacterial pathogens. Bacterial drug resistance is the phenomenon that after repeated contact with drugs, the susceptibility of pathogens to the drugs decreases or even disappears, resulting in reduced or insufficient efficacy of these drugs against infections [4]. According to the Centers for Disease Control and Prevention, at least 2 million people are infected with drug-resistant bacteria each year in the United States, killing at least 23,000 people [5]. Drug resistance has recently become a widespread problem in medical care, and the increase in drug-resistant infections is much faster than the development of new drugs approved for use in humans. Therefore, it is imperative to develop new antimicrobial agents.

Natural products are an important source of new and highly effective antibacterial agents, which can be used in the fight against the growing drug resistance due to the emergence of multidrug- and extensively drug-resistant bacterial phenotypes [6,7]. Among them are natural alkaloids, which are a group of basic, nitrogen-containing organic compounds with significant biological activities. Alkaloids are critical for the effects of many Chinese herbal medicines. Based on their different chemical structures, alkaloids can be divided into isoquinolines, pyrroles, pyridines, quinolines, indoles, and ten other types of alkaloids [8]. Several in vivo and clinical investigations have reported that alkaloids have various pharmacological effects, including anticancer [9], antiviral [10], anti-inflammatory [11], and antibacterial activities [12]. Due to their extensive pharmacological activities, many alkaloid drugs have been developed, such as morphine as a narcotic analgesic [13], the cough medicine codeine [14], the anticancer drug vinblastine [15], and antibacterial drug berberine hydrochloride [16], all of which are widely used in clinical practice. In recent years, the antibacterial activity of alkaloids has been extensively assessed in biomedical investigations. Studies on the antibacterial mechanism of natural alkaloids show that they can disrupt the bacterial cell membrane [17], affect the DNA function [18], and inhibit protein synthesis [19]. Thus, natural alkaloids are potentially active against various bacteria, including methicillin-resistant *Staphylococcus aureus* (MRSA) [20], which is a common causative agent of infections in the clinic. They are being used as lead compounds for the development of new antimicrobial drugs.

As a group of promising natural antibiotics, the alkaloids can be recovered from many natural sources, and they have a wide antibacterial spectrum with a good antibacterial effect on common clinical strains, including drug-resistant bacteria. When we performed an in-depth literature search, we did not find a comprehensive review on the antibacterial activity and mechanism of alkaloids. This article reviews the chemical structure, antibacterial activity, and mechanism of alkaloids reported in recent years. We propose solutions to the current problems associated with the use of alkaloids as antibacterial agents, and we provide the theoretical basis for the development of new antibacterials derived from natural products.

## 2. Antibacterial Activity of Different Types of Natural Alkaloids

As natural products, alkaloids are widely distributed in nature [21]. In recent years, due to the seriousness of bacterial drug resistance, the antibacterial activity of alkaloids has received widespread attention [22]. This paper focuses on the bacteriostatic effects of isoquinoline alkaloids, pyridine alkaloids, indole alkaloids, steroidal alkaloids, and other alkaloids, among which indole alkaloids and isoquinoline alkaloids represent the main types of compounds with antibacterial activity [8].

### 2.1. Isoquinoline Alkaloids

Isoquinoline alkaloids, also known as benzylisoquinoline alkaloids, are characteristic plant-specific metabolites with an isoquinoline skeleton that have a long history of research [23]. They are widely distributed in Papaveraceae, Berberidaceae, Ranunculaceae, and Menispermaceae. They form the largest alkaloid group that can be further divided into subgroups, including simple isoquinoline alkaloids, benzylisoquinoline alkaloids, bisbenzylisoquinoline alkaloids, aporphine alkaloids, and protoberberine alkaloids [8].

Thalicfoetine, which was isolated from the roots of *Thalictrum foetidum*, is an isoquinoline alkaloid with a spiro tetrahydropyridine-furanone core. In an antibacterial activity test using the broth dilution method, thalicfoetine significantly inhibited *Bacillus subtilis* at a minimum inhibitory concentration (MIC) of 3.12 µg/mL, and the MIC of the antibiotic cefotaxime against *Bacillus subtilis* was 1.56 μg/mL. Thus, the inhibitory activity of thalicfoetine was comparable to that of cefotaxime [24].

In a search for new natural products with antibacterial effect, spathullin A and B were recovered from a culture broth of *Penicillium spathulatum* Em19. Both compounds were active against Gram-negative and Gram-positive bacteria, including *Enterobacter cloacae*, *Escherichia coli*, *Pseudomonas aeruginosa*, *Acinetobacter baumannii*, *Klebsiella pneumonia*, and *S. aureus*. Spathullin B was more effective against all tested bacterial pathogens than spathullin A, with an MIC of 1 µg/mL against *S. aureus.* The MICs of meropenem and ciprofloxacin against *S. aureus* were 0.5 and 0.25 μg/mL, respectively. These results showed that spathullin B had a strong antibacterial activity [25].

An assessment of the antibacterial activity of isoquinoline alkaloids isolated from *Chelidonium majus* showed that chelerythrine (CHE) was the most effective against *P. aeruginosa* (MIC, 1.9 µg/mL), and sanguinarine against *S. aureus* (MIC, 1.9 µg/mL). CHE and chelidonine exerted strong antifungal activity against *Candida albicans* [26].

These alkaloids show good antibacterial activity by inhibiting Gram-negative bacteria, Gram-positive bacteria, and fungi. Their antibacterial effect is similar to that of commonly used antimicrobials on the market.

### 2.2. Pyridine Alkaloids

Nitrogen-containing pyridine alkaloids are derived from pyridine or piperidine. They can be divided into simple pyridine alkaloids, quinolizidine alkaloids, and indolizidine alkaloids [8]. These alkaloids are widely distributed in Palmaceae, Leguminosae, Zingiberaceae, Solanaceae, and other plant groups, and their bacteriostatic or bactericidal activities have been widely studied.

A study on the antimicrobial activity of an Amaryllidaceae extract showed that caranine was active against *Candida dubliniensis* with an MIC of 128 μg/mL. The control antibiotic tetracycline had no antimicrobial activity against *C. dubliniensis*, but caranine can be used as a new antifungal drug [27].

Twelve quinoline glycoside alkaloids were isolated from *Sophora tonkinensis*. Antibacterial activity screening identified compounds lanatine A, cermizine C, jussiaeiine A, jussiaeiine B, 3-(4-hydroxyphenyl)-4-(3-methoxy-4-hydroxyphenyl)-3,4-dehydroquin-olizidine, and (−)-*N*-methyl-cytisine with inhibitory activity against *S. aureus* and *E. coli* with an MIC range of 0.8–18.0 μg/mL [28].

The structure of an alkaloid isolated from *Zingiberis rhizoma* was elucidated based on 1D and 2D NMR spectra, along with MS spectra. The identified compound 2-methoxy-4-(2-(2-pyridine)-ethyl) phenol exhibited substantial bioactivity against *C. albicans* ATCC 10231 with an MIC of 1.0 mg/mL [29].

Pyridine alkaloids have good antimicrobial activity against a variety of pathogens, indicating that they can be used as broad-spectrum antimicrobial agents.

### 2.3. Indole Alkaloids

Indole alkaloids are tryptophan derivatives with many complex structures and significant biological activities. They are mainly distributed in Loganaceae, Apocynaceae, Rubiaceae Juss., and other plant groups. Based on their core structures, they are mainly divided into monomeric indole alkaloids, tryptamine indole alkaloids, monoterpenoid indole alkaloids, and bis-indolic alkaloids [8,12].

A strychnine alkaloid, 16,17,19,20-tetrahydro-2,16-dehydro-18-deoxyisostrychnine, was isolated from the leaves of *Psychotria pilifera*. In a bacteriostatic activity assay against *E. coli* ATCC 11775, *S. aureus* ATCC 25922, and *Enterococcus faecalis* ATCC 10541, the new alkaloid exhibited selective antibacterial activity against *E. coli*, which was equivalent to cefotaxime with an MIC of 0.781 μg/mL [30].

The deep sea-derived *Streptomyces* sp. SCSIO 11791 produces two chlorinated bis-indole alkaloids, dionemycin and 6-CH_3_O-7,7’-dichorochromopyrrolic acid. Dionemycin was tested for antibacterial activity against six MRSA strains isolated from humans and pigs. The MICs of dionemycin were in the range of 0.5–2 μg/mL. The MIC range of the positive control kanamycin was 8–128 μg/mL. Dionemycin showed good antibacterial activity [31].

Two monoterpene indole alkaloids, the voacafricines A and B, were isolated from the fruit of *Voacanga africana* and tested for antibacterial activity. The MIC of voacafricine A against *S. aureus* was 3.12 μg/mL. The antibacterial activity of voacafricine B was stronger than that of voacafricine A. The MICs of voacafricine B against *S. aureus* and *Salmonella typhimurium* were 3.12 and 0.78 µg/mL, respectively. The MICs of the positive control alkaloid berberine against *S. aureus* and *S. typhimurium* were 6.25 and 3.12 μg/mL, respectively. The MICs of the positive control alkaloid fibraurtine against *S. aureus* and *S. typhimurium* were 25 and 3.12 μg/mL, respectively. Thus, these two voacafricines have a strong antibacterial activity, which is superior to that of the well-studied alkaloids berberine and fibraurtine [32].

MRSA is resistant to a variety of antimicrobials on the market, but indole alkaloids have strong antibacterial activity against MRSA and can be developed as therapeutics against this type of drug-resistant infection.

### 2.4. Steroidal Alkaloids

Steroidal alkaloids are nitrogen-containing derivatives of natural steroids, with most of the nitrogen atoms localized in the ring structure [8]. These alkaloids are found in plants, including Solanaceae, Liliaceae, Apocynaceae, and Buxaceae, as well as in amphibians and marine invertebrates, and they are typically isolated as glycoalkaloids [33,34,35]. Steroidal alkaloids are divided into three categories based on the steroid skeleton: pregnane alkaloids, cyclopregnane alkaloids, and cholestane alkaloids [28].

The seeds of *Holarrhena antidysenteriaca* Wall. ex A.DC. contain two steroidal alkaloids, isoconkuressine and *N*-formylconessimine. Their intrinsic antibacterial activities were tested against methicillin-sensitive *S. aureus* (MSSA) and MRSA. The results showed that the newly identified steroidal alkaloids had potent antibacterial activity [36].

The steroid compound combretin was isolated from the seeds of *Combretum quadrangulare* Kurz. The MIC of the purified compound was determined using the two-fold dilution method. The results showed that the antibacterial activity of combretin against *E. coli* ATCC 25922 and *P. aeruginosa* ATCC 27853 was better than that against *S. aureus* ATCC 25923 [37].

Three steroidal alkaloids, mokluangin A, B, and C, were extracted from *Holarrhena pubescens*. The antibacterial activity tests of the alkaloid extracts showed that mokluangin B was moderately active against *B. subtilis* and *E. coli* with an MIC of 16 µg/mL, while mokluangin C exhibited a selective, moderate activity against *E. coli* with an MIC of 16 µg/mL. Vancomycin and gentamycin were used as standard drugs with MIC values in the range of 0.125–0.25 µg/mL. Although these steroidal alkaloids have moderate antibacterial activity, they are still suitable as positive control compounds [38].

In recent years, an increasing number of studies showed that steroidal alkaloids have good antibacterial activity. Moreover, it was also found that steroidal alkaloids could be used in combination with antibiotics to enhance the activity of these drugs [39]. This provides a new direction for the development of antibiotics.

### 2.5. Others

Alkaloids have been gradually applied in clinical practice, and their effects are relatively significant. In addition to the compounds described in the preceding subsections, antibacterial properties have also been identified in quinoline alkaloids, piperidine alkaloids, polyamine alkaloids, imidazole alkaloids, and peptide alkaloids [8].

Epidihydropinidine, which is the main piperidine alkaloid in Norway spruce, has promising antibacterial and anti-*Candida* activities. We showed in our earlier investigation that this alkaloid exerted growth-inhibitory activity against a range of bacterial and fungal strains, showing an MIC of 5.37 µg/mL against *P. aeruginosa*, *E. faecalis*, *Candida glabrata*, and *C. albicans*. The positive controls tetracycline, ampicillin, and amphotericin B had almost no effect on these bacteria. Epidihydropinidine shows good antibacterial activity and has a wide antibacterial spectrum [40].

Myoporumine A and myoporumine B are two alkaloids isolated from the semi-mangrove plant *Myoporum bontioides* A. Gray. These two alkaloids displayed a strong anti-MRSA activity with an MIC of 6.25 µg/mL. The MIC of the positive control vancomycin was 0.78 μg/mL. It is suggested that these two compounds can be used as new anti-MRSA drugs [41].

Eight known alkaloids were isolated from the organic extracts of the sponge *Agelas dilatata*. Antibacterial activity testing of these alkaloids identified bromoageliferin with significant activity against *P. aeruginosa*. The findings confirmed bromoageliferin as a potential lead compound for designing new antibacterial drugs [42].

These studies show that alkaloid compounds have a wide range of antibacterial activities with a broad antibacterial spectrum that includes activity against *Helicobacter pylori*, *P. aeruginosa*, *E. faecalis*, *S. aureus*, *C. glabrata*, and *C. albicans*. The antibacterial activities of natural alkaloids are presented in Table 1.

**Table 1:** Antibacterial activities of natural alkaloids.

Alkaloids (Compound Name)	Sources	Structure of the Alkaloids	Strains Inhibited	MIC *	Positive Control	MIC *	References
2-methoxy-4-(2-(2-pyridine)-ethyl) phenol	*Zingiberis rhizoma*	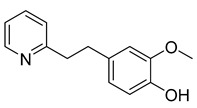	*C. albicans*	1.0 mg/mL	-	-	[29]
Thalicfoetine	*T. foetidum*	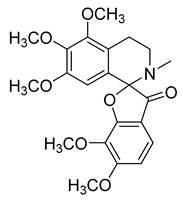	*B. subtilis*	3.12 µg/mL	Cefotaxime	1.56 µg/mL	[24]
Spathullin A	*P. spathulatum* Em19	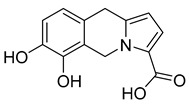	*E. coli*, *A. baumannii*, *E. cloacae*, *S. aureus*	4–15 µg/mL	Meropenem	0.5–2 µg/mL	[25]
Spathullin B	*P. spathulatum* Em19	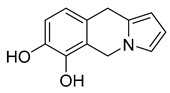	*E. coli*, *A. baumannii*, *E. cloacae*, *S. aureus*	1–15 µg/mL			[25]
16,17,19,20-tetrahydro-2,16-dehydro-18-deoxyisostrychnine	*P. pilifera*	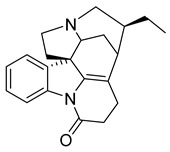	*E. coli*	0.781 μg/mL	-	-	[30]
tTris(1H-indol-3-yl) methylium	*Pseudomonas aeruginosa* UWI-1	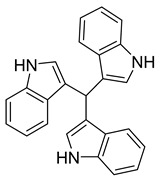	Gram-positive bacteria, Gram-negative bacteria	1–128 µg/mL	Kanamycin	4–128 µg/mL	[43]
bis(indol-3-yl) phenylmethane	*P. aeruginosa* UWI-1	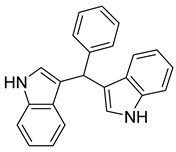	Gram-positive bacteria	32–128 µg/mL			[43]
indolo (2,1b) quinazoline-6,12 dione	*P. aeruginosa* UWI-1	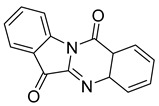	Gram-positive bacteria, Gram-negative bacteria	1–32 µg/mL			[43]
Dionemycin	*Streptomyces* sp. SCSIO 11791	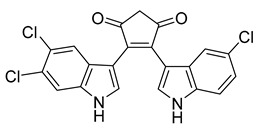	*M. luteus*, *S. aureus*, MRSA	0.5–2 μg/mL	Kanamycin	1–128 μg/mL	[31]
6-CH_3_O-7′,7′′-dichorochromopyrrolic acid	*Streptomyces* sp. SCSIO 11791	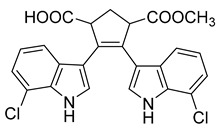	*M. luteus*, *S. aureus*, MRSA	3–128 μg/mL			[31]
Voacafricines A	*V. africana*	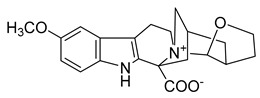	*S. aureus*, *S. typhi*, *B. Subtilis*	3.12–25 μg/mL	Berberine, Fibraurtine	3.12–25 μg/mL	[32]
Voacafricines B	*V. africana*	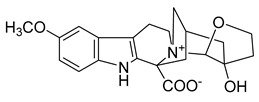	*S. aureus*, *S. typhi*, *B. Subtilis*, *E. coli*	0.78–50 μg/mL			[32]
Epidihydropinidine	*Picea abies* (L.) Karsten	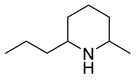	*P. aeruginosa*, *E. faecalis*, *C. glabrata*, *C. albicans*, *S. enterica*, *B. cereus*, *S. aureus*	5.37–43 µg/mL	Amphotericin B	-	[40]
Myoporumine A	*M. bontioides* A. Gray	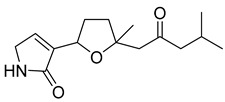	MRSA	6.25 µg/mL	Fraction F4	25 µg/mL	[41]
Myoporumine B	*M. bontioides* A. Gray	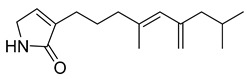	MRSA	6.25 µg/mL			[41]
Palmatine	*Coptis chinensis*	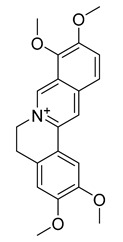	*H. pylori*	75–200 μg/mL	Metronidazole	0.5–2 μg/mL	[44]
Chelerythrine	*Toddalia asiatica* (Linn) Lam	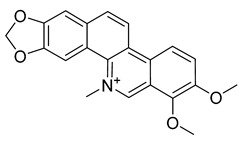	*S. aureus*, MRSA, Extended-spectrum *β*-lactamases *S. aureus*	0.156 mg/mL	-	-	[45]
Berberine	*T. asiatica* (Linn) Lam	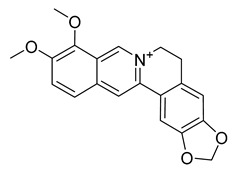	*S. aureus*, MRSA, Extended-spectrum *β*-lactamases *S. aureus*	0.0312 mg/mL	-	-	[45]
Tetrandrine	*Stephania tetrandra* S. Moore	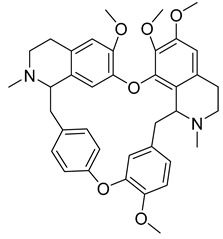	*S. aureus*	125–250 μg/mL	Ampicillin, Oxacillin	0.9–250 μg/mL	[46]
Stachydrine	*Ritchiea capparoides* var. *longipedicellata*	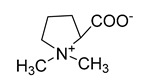	*S. aureus*, *E. coli*	5 mg/mL	Streptomycin	0.125 mg/mL	[47]
Chabamide	*Piper chaba*	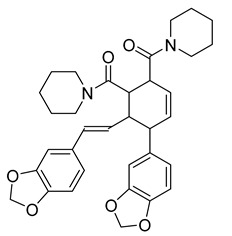	*M. tuberculosis*	12.5 μg/mL	-	-	[48]
Lycorine	*Pancratium Foetidum* Pom	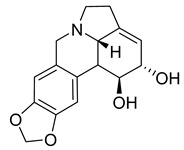	*S. aureus*, *B. cereus*, *P. aerugin*, *E. cloac*	0.24 mg/mL	Streptomycin	0.04–0.34 mg/mL	[49]
Lycorine	*Amaryllidaceae*	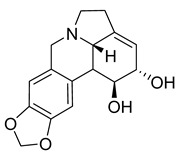	*C. dubliniensis*, *C. albicans*, *L. elongisporus*	32–64 µg/mL	Tetracycline	0.5–2 µg/mL	[27]
Caranine	*Amaryllidaceae*	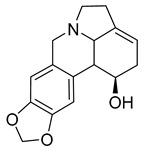	*C. dubliniensis*	128 µg/mL			[27]
6-(pyrrolidin-2-yl)DAPG	*Pseudomonas protegens* UP46	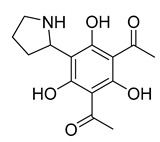	*S. aureus*, *Bacillus cereus*	2–4 µg/mL	-	-	[50]
6-(piperidin-2-yl)DAPG	*P. protegens* UP46	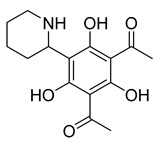	*S. aureus*, *B. cereus*	2 µg/mL	-	-	[50]
Kopsiahainanins A	*Kopsia hainanensis*	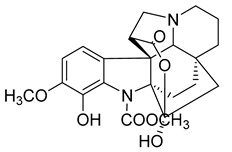	*S. aureus*, *S. epidermidis*, *E. coli*, *E. cloacae*, *K. pneumoniae*, *P. aeruginosa*, *S. dysenteriae*	0.12–0.23 µg/mL	Netilmicin	0.004–0.015 µg/mL	[51]
Kopsiahainanins B	*K. hainanensis*	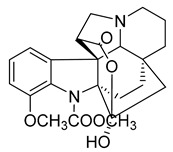	*S. aureus*, *S. epidermidis*, *E. coli*, *E. cloacae, K. pneumoniae*, *P. aeruginosa*, *S. dysenteriae*	0.14–0.26 µg/mL			[51]
Kopsiahainanins C	*K. hainanensis*	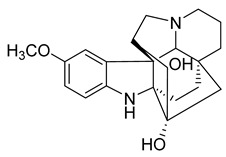	*S. aureus*, *S. epidermidis*, *E. coli*, *E. cloacae*, *K. pneumoniae*, *P. aeruginosa*, *S. dysenteriae*	0.94–1.32 µg/mL			[51]
Kopsiahainanins D	*K. hainanensis*	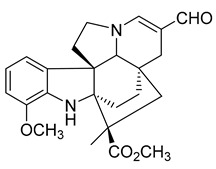	*S. aureus*, *S. epidermidis*, *E. coli*, *E. cloacae*, *K. pneumoniae*, *P. aeruginosa*, *S. dysenteriae*	0.92–1.24 µg/mL			[51]
Kopsiahainanins E	*K. hainanensis*	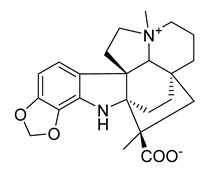	*E. coli*, *E. cloacae, K. pneumoniae*, *P. aeruginosa*, *S. dysenteriae*	1.19–1.31 µg/mL			[51]
Kopsiahainanins F	*K. hainanensis*	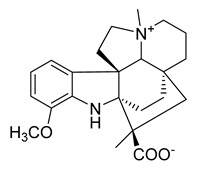	*E. coli*, *E. cloacae*, *K. pneumoniae*, *P. aeruginosa*, *S. dysenteriae*	0.99–1.32 µg/mL			[51]
Kuanoniamine D	Ascidian *Cystodytes dellechiajei*	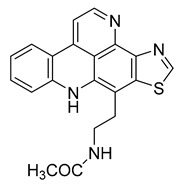	*E. coli*, *M. luteus*	2.2–17.4 µM	Gentamicin	0.02–0.08 µM	[52]
Shermilamine B	Ascidian *C. dellechiajei*	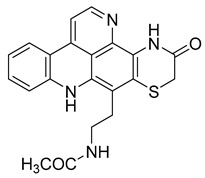	*E. coli*, *M. luteus*	2.0–8.0 µM			[52]
*N*-deacetylkuanoniamine D	Ascidian *C. dellechiajei*	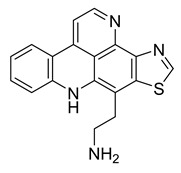	*E. coli*, *M. luteus*	2.5 µM			[52]
*N*-deacetylshermilamine B	Ascidian *C. dellechiajei*	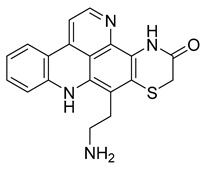	*E. coli*, *M. luteus*	1.1–4.5 µM			[52]
11-hydroxyascididemin	Ascidian *C. dellechiajei*	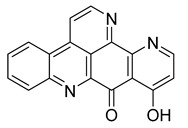	*E. coli*, *M. luteus*	2.6–10.5 µM			[52]
Cystodimine A	Ascidian *C. dellechiajei*	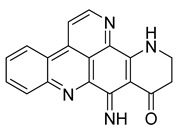	*E. coli*, *M. luteus*	1.2–2.4 µM			[52]
Cystodimine B	Ascidian *C. dellechiajei*	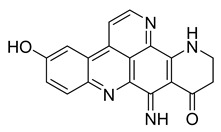	*E. coli*, *M. luteus*	2.6–10.5 µM			[52]
Ascididemin	Ascidian *C. dellechiajei*	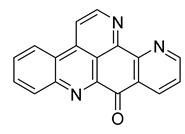	*E. coli*, *M. luteus*	0.2–0.3 µM			[52]
Sophoridine	*Thermopsis lanceolata* R.Brown	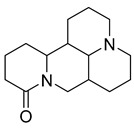	*E. coli*, *E. aerogenes*, *P. vulgaris*, *B. subtilis*, *S. epidermidis*	2 × 10^−2^–4 × 10^−2^ M	-	-	[53]
Sophoramine	*T. lanceolata* R.Brown	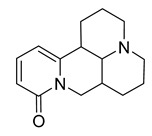	*E. coli*, *E. aerogenes*, *P. vulgaris*, *B. subtilis*, *S. epidermidis*	4 × 10^−2^–5 × 10^−2^ M	-	-	[53]
Matrine	*T. lanceolata* R.Brown	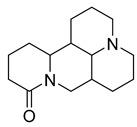	*E. coli*, *E. aerogenes*, *P. vulgaris*, *B. subtilis*, *S. epidermidis*	2 × 10^−2^–5 × 10^−2^ M	-	-	[53]
Cytisine	*T. lanceolata* R.Brown	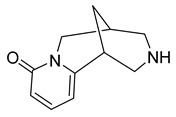	*E. coli*, *E. aerogenes*, *P. vulgaris*, *B. subtilis*, *S. epidermidis*	3 × 10^−2^–5 × 10^−2^ M	-	-	[53]
Oxymatrine	*T. lanceolata* R.Brown	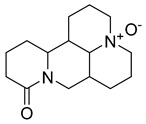	*E. coli*, *E. aerogenes*, *P. vulgaris*, *B. subtilis*, *S. epidermidis*	5 × 10^−2^ M	-	-	[53]
Berberine	*Berberis vulgaris*	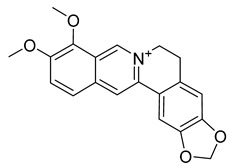	*T. mentagrophytes*, *T. rubrum*, *M. canis*, *M. gypseum*	0.062–0.250 mg/mL	Ketoconazole	0.125–0.250 mg/mL	[54]
Chelerythrine	*C. majus*	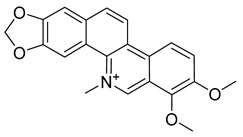	*P. aeruginosa*	1.9 mg/L	-	-	[26]
Sanguinarine	*C. majus*	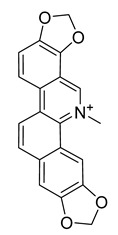	*S. aureus*	1.9 mg/L	-	-	[26]
Chelidonine	*C. majus*	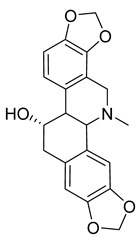	*C. albicans*	62.5 mg/L	-	-	[26]
Berberine	*C. majus*	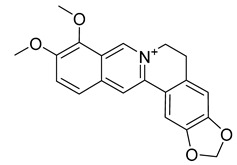	*S. aureus*	125 mg/L	-	-	[26]
Allocryptopine	*C. majus*	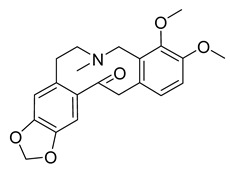	*S. aureus*	125 mg/L	-	-	[26]
Mokluangins B	*Holarrhena pubescens*	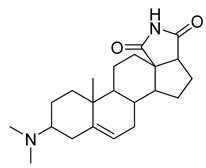	*B. subtilis*, *E. coli*	16 µg/mL	Vancomycin, Gentamycin	0.125–0.25 µg/mL	[38]
Mokluangins C	*H. pubescens*	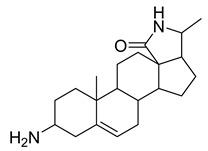	*E. coli*	16 µg/mL			[38]
Ageliferin	Sponge *A. dilatata*	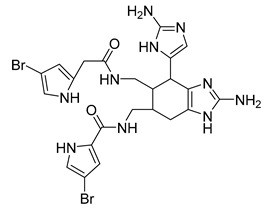	*A. baumannii*, *K. pneumoniae*, *P. aeruginosa*	64 ≥ 128 mg/L	-	-	[42]
Bromoageliferin	Sponge *A. dilatata*	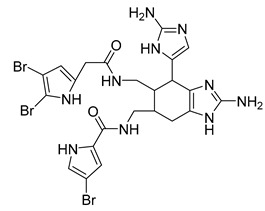	*A. baumannii*, *K. pneumoniae*, *P. aeruginosa*	8 ≥ 128 mg/L	-	-	[42]
Dibromoageliferin	Sponge *A. dilatata*	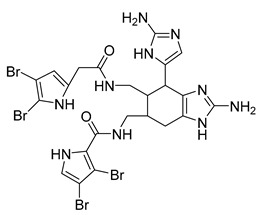	*A. baumannii*, *K. pneumoniae*, *P. aeruginosa*	32 ≥ 128 mg/L	-	-	[42]
Sceptrin	Sponge *A. dilatata*	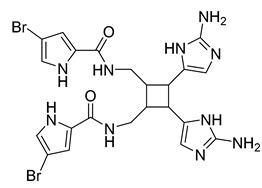	*A. baumannii*, *K. pneumoniae*, *P. aeruginosa*	64 ≥ 128 mg/L	-	-	[42]
Nakamuric acid	Sponge *A. dilatata*	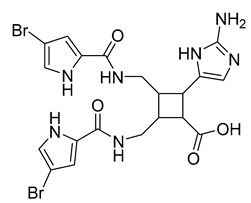	*A. baumannii*, *K. pneumoniae*, *P. aeruginosa*	≥128 mg/L	-	-	[42]
4-Bromo-1H-pyrrole-2-carboxylic acid	Sponge *A. dilatata*	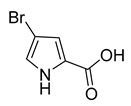	*A. baumannii*, *K. pneumoniae*, *P. aeruginosa*	64 ≥ 128 mg/L	-	-	[42]
4,5-Dibromopyrrole-2-carboxylic acid	Sponge *A. dilatata*	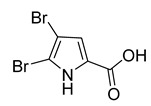	*A. baumannii*, *K. pneumoniae*, *P. aeruginosa*	64 ≥ 128 mg/L	-	-	[42]
3,7-Dimethylisoguanine	Sponge *A. dilatata*	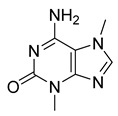	*A. baumannii*, *K. pneumoniae*, *P. aeruginosa*	64 ≥ 128 mg/L	-	-	[42]
9H-carbazole	Myxobacterium *L. luteola*	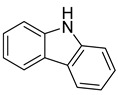	*C. albicans*, *B. subtilis*, *E. coli*, *C. violaceum*	6.7–33.3 µg/mL	Methanol	-	[55]
3-chloro-9H-carbazole	Myxobacterium *L. luteola*	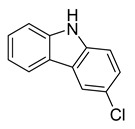	*C. albicans*	33.3 µg/mL			[55]
4-hydroxymethyl-quinoline	Myxobacterium *L. luteola*	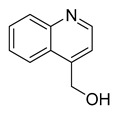	*C. albicans*	33.3 µg/mL			[55]
Latifolianine A	*Nauclea latifolia*	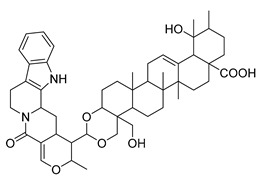	*H. influenzae*	25 µg/mL	Ciprofloxacin	1.6 µg/mL	[56]
Latifoliaindole A	*N. latifolia*	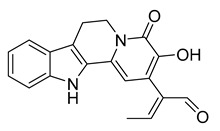	*H. influenzae*	50 µg/mL			[56]
Latifoliaindole B	*N. latifolia*	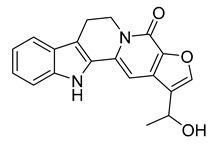	*H. influenzae*	25			[56]
Neoechinulin A	*Eurotium* sp.	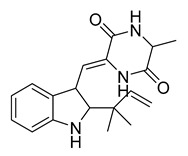	*B. cereus*, *P. vulgaris*	6.25–25 μM	Ciprofloxacin	0.20–0.78 μM	[57]
l-alanyl-l-tryptophan anhydride	*Eurotium* sp.	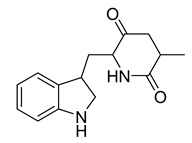	*B. cereus*, *P. vulgaris*	1.56–3.13 μM			[57]
Dihydroxyisoechinulin A	*Eurotium* sp.	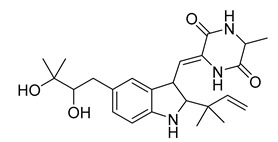	*B. cereus*	3.13 μM			[57]
Terpendole L	*Tolypocladium* sp.	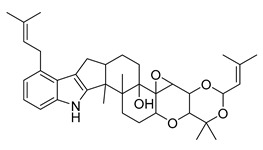	*M. lysodeikticus*, *M. luteus*	6.25–50 μg/mL	Ciprofloxacin	0.78 μg/mL	[58]
Tolypocladin A	*Tolypocladium* sp.	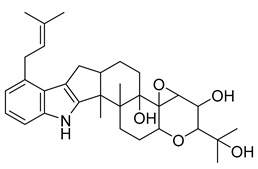	*B. cereus*, MRSA	12.5–25 μg/mL	Ketoconazole	0.78 μg/mL	[59]
Tolypocladin B	*Tolypocladium* sp.	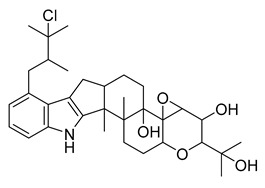	MRSA	50 μg/mL			[59]
Tolypocladin H	*Tolypocladium* sp.	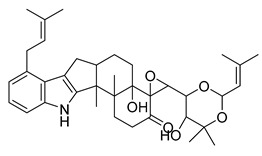	*B. cereus*, *M. lysodeikticus*, *B. paratyphosum*, *B. subtilis*, *E. aerogenes*, *S. typhi*, *P. vulgaris*	0.78–1.56 μg/mL			[59]
Alstoniascholarine A	*Alstonia scholaris*	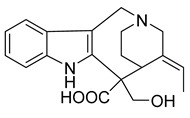	*P. aeruginosa*, *K. pneumoniae*, *E. coli*, *E. faecalis*	25–50 μg/mL	Gentamycin, Griseofulvin	0.20–7.81 μg/mL	[60]
Alstoniascholarine C	*A. scholaris*	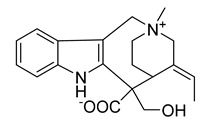	*P. aeruginosa*, *K. pneumoniae*, *E. coli*, *E. faecalis*	12.5–50 μg/mL			[60]
Alstoniascholarine E	*A. scholaris*	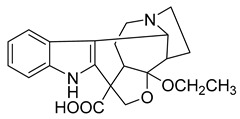	*P. aeruginosa*, *K. pneumoniae*, *E. coli*, *E. faecalis*	25–50 μg/mL			[60]
Alstoniascholarine F	*A. scholaris*	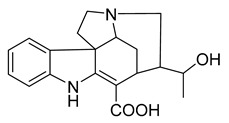	*P. aeruginosa*, *K. pneumoniae*, *E. coli*, *E. faecalis*	3.13–50 μg/mL			[60]
Alstoniascholarine H	*A. scholaris*	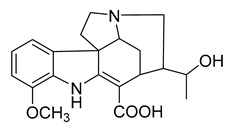	*P. aeruginosa*, *K. pneumoniae*, *E. coli*	25–50 μg/mL			[60]
Alstoniascholarine I	*A. scholaris*	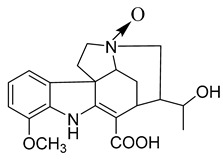	*P. aeruginosa*, *K. pneumoniae*, *E. coli*	12.5–25 μg/mL			[60]
Alstoniascholarine J	*A. scholaris*	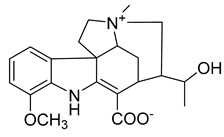	*S. aureus*, *P. aeruginosa*, *E. faecalis*, *K. pneumoniae*, *E. coli*	3.13–25 μg/mL			[60]
Scholarisine T	*A. scholaris*	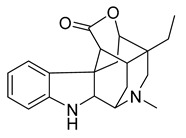	*E. coli*, *B. subtilis*, *S. typhi*	0.78–12.5 μg/mL	Cefotaxime	0.39–3.12 μg/mL	[61]
Scholarisine U	*A. scholaris*	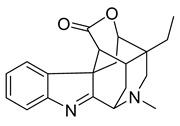	*E. coli*, *B. subtilis*	0.78–3.12 μg/mL			[61]
Scholarisine V	*A. scholaris*	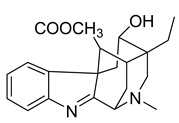	*E. coli*, *S. aureus*, *S. typhi*	0.78–12.5 μg/mL			[61]
2, 5, 6-tribromo-3-[(3′-bromo-4′-hydroxyl-phenyl)-methyl]-1H-indole	*Laurencia similis*	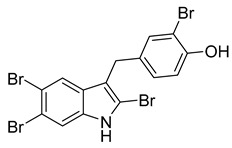	*B. subtilis*, *B. thuringensis*, *S. aureus*, *A. tumefaciens*, *P. lachrymans*, *R. solanacearum*, *X. vesicatoria*	2–8 μg/mL	Penicillin	0.125–250 μg/mL	[62]
5, 6-dibromo-1-hydroxy-3-isopropenyl-indole-2-one	*L. similis*	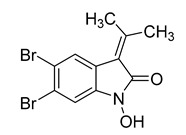	*S. aureus*, *A. tumefaciens*	12.5 μg/mL			[62]
Neofiscalin A	*Neosartorya siamensis* KUFA 0017	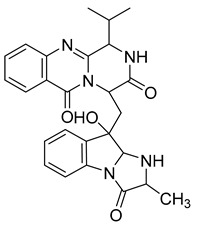	MRSA, VRE	8 μg/mL	Oxacillin	128 μg/mL	[63]
Dragmacidin G	Genus *Spongosorites*	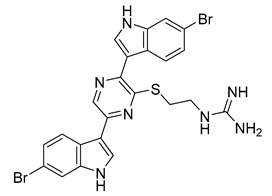	*S. aureus*, MRSA	0.62 µg/mL	Chloramphenicol	3.1–6.2 µg/mL	[64]
Chaetoglobinol A	*Chaetomium globosum*	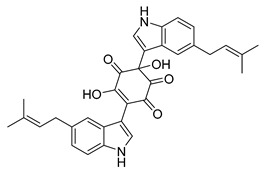	*B. subtilis*	50 µg/mL	-	-	[65]
Phutdonginin	*Kopsia arborea* Blume	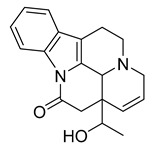	*E. coli*	32 g/mL	-	-	[66]

* MIC (minimum inhibitory concentration) is the lowest drug concentration at which a given antifungal extract inhibits the visible growth of a tested organism.

## 3. Antibacterial Mechanisms of Natural Alkaloids

The underlying mechanisms of the antibacterial activities of alkaloids have been gradually discovered in relation to their unique chemical structures [67]. Alkaloids inhibit bacterial growth through a variety of mechanisms, including inhibition of the bacterial nucleic acid and protein synthesis, modification of the bacterial cell membrane permeability, damage of the cell membrane and cell wall, inhibition of bacterial metabolism [68], and inhibition of efflux pumps.

### 3.1. Inhibition of Bacterial Nucleic Acid and Protein Synthesis

Bacterial nucleic acids consist of DNA and RNA. DNA molecules store, copy, and transmit genetic information, and RNA molecules function as messenger molecules to ensure proper protein synthesis [69]. Therefore, damage of DNA/RNA molecules or inhibition of DNA replication prevents the expression of virulence genes, which affects the traits of microorganisms and their growth and reproduction [70]. The filamentous temperature-sensitive protein Z (FtsZ) plays an important role in the process of bacterial cell division, participates in the formation of diaphragms, and forms a ring structure at the division site, which ultimately controls the process of bacterial cell division [71]. Therefore, FtsZ is also a target for the screening of new antimicrobial agents.

In one study, researchers used *Galleria mellonella* as an infection model to report, for the first time, that berberine also has certain anti-invasive activity in vitro [72]. Studies have shown that berberine inhibits DNA replication, RNA transcription, and protein biosynthesis in bacteria. Driven by membrane potential and accumulation in cells, it is an excellent DNA intercalator [73]. The binding of berberine to DNA and RNA changes the structure of these macromolecules and can potentially damage or break their strands so that they are no longer normal templates for DNA replication, RNA transcription, and protein biosynthesis [74]. Animal and clinical studies have demonstrated that berberine has low toxicity and few side effects [75], and in vitro toxicity studies showed that it has no significant genotoxic, mutagenic, or cytotoxic activity [76,77].

Antibacterial testing of CHE revealed a strong inhibitory activity against Gram-positive bacteria, such as *S. aureus*, including MRSA and extended-spectrum *β*-lactamase (ESBL)-producing *S. aureus*. The effect of alkaloids on protein expression was assessed by SDS-PAGE. The results showed that the amount of protein leakage was positively correlated with the concentration of CHE. It was obvious that the damaging effect of CHE on the bacterial cells resulted in the elimination of soluble protein, thus offsetting the proportion of protein consumed by the bacterial cells. Compared with a control group, CHE effectively inhibited the protein expression in MRSA cells within 3 h. The inhibitory effect of CHE decreased with time, which might have been due to the soluble protein in the culture supernatant that the surviving bacteria were still consuming to support growth. However, this could also be due to bacterial infection and activation of self-repair mechanisms, which directly led to the reduction in protein leakage [45]. However, the cytotoxicity of CHE has yet to be evaluated in clinical studies.

The FtsZ protein is essential for the cell cycle and, especially, the cell division in *E. coli* [78]. It participates in diaphragm formation and forms a ring structure at the division site to control the bacterial cell division process. Sanguinarinerine strongly induces filamentation in both Gram-positive and Gram-negative bacteria and prevents bacterial cell division by inhibiting cytokinesis. It perturbs the cytokinetic Z-ring formation in *E. coli.* Moreover, sanguinarinerine inhibits the assembly of purified FtsZ and reduces the bundling of FtsZ protofilaments in vitro [79]. Similarly, matrine inhibits the synthesis of proteins related to cell growth and division in *E. coli* and *S. aureus*, ultimately blocking the division and growth of bacteria. Matrine binds to proteins in cells, forming aggregates, leading to the disintegration of the cytoplasm, which eventually kills the bacteria. The MICs of matrine against *E. coli* and *S. aureus* were 2.5 and 10 mg/mL, respectively [80]. Berberine inhibits *E. coli* by negatively regulating the FtsZ protein [81]. Indole alkaloids can be used as potential drug resistance reversal agents that exert antibacterial activity by inhibiting FtsZ and MRSA pyruvate kinase [12].

### 3.2. Effect on the Bacterial Cell Membrane Permeability—Damage of Cell Membrane and Cell Wall

The bacterial cell membrane is an elastic, semipermeable membrane composed of phospholipid bilayers and proteins. It provides a relatively stable internal environment for bacterial life activities and plays a role in cell recognition and electron transfer. The ability to form biofilms provides a selective advantage for bacterial survival under harsh environmental conditions [82]. Severe damage to the bacterial cell membrane breaks the protective barrier of the cell and causes the release of a large number of molecules. Furthermore, the defense function of the cell wall is lost, and the transport function and the information transfer function of the cell membrane are blocked. Specifically, the leakage of intracellular electrolytes into the culture medium increases the conductivity of the medium. Therefore, variations in the culture supernatant conductivity can indicate changes in the bacterial cell membrane permeability [83]. Alkaline phosphatase (AKP) mainly resides between the bacterial cell wall and membrane. AKP leaks out of cells due to increases in the permeability of the bacterial cell wall. AKP activity measurements can indirectly reflect the integrity of the bacterial cell wall [84,85].

The MIC of sanguinarine against *Providencia rettgeri* was determined by the agar dilution method, and the effect of sanguinarine on biofilm formation was detected by laser scanning confocal microscopy (CLSM), field emission scanning electron microscopy (FESEM), and crystal violet staining. Sanguinarinerine had an MIC of 7.8 mg/mL against *P. rettgeri*. The compound inhibited the growth of *P. rettgeri* and destroyed the cell membrane integrity. The CLSM, FESEM, and crystal violet staining results also showed that sanguinarine had a significant inhibitory effect on the *P. rettgeri* biofilm by blocking the expression and formation of biofilm substances, which led to the inactivation of *P. rettgeri* [68]. By changing the quorum sensing system, researchers observed that berberine inhibited biofilm formation in drug-resistant *E. coli* strains [86].

Alkaloids from *Dicranostigma leptopodum* (Maxim) displayed a substantial antibacterial effect against *K. pneumoniae* with an MIC of 3.0 mg/mL. The conductivity of the culture medium treated with the alkaloids of *Dicranostigma leptopodum* was significantly higher than that of the control group. Specifically, with the extension of the action time, the conductivity of the culture medium showed a trend of first increasing and then stabilizing, indicating that the damage of the cell membrane by the inhibitor increased the conductivity of the culture medium. Breaking the protective barrier by cell membrane damage leads to electrolyte leakage into the culture medium, causing a change in the conductivity of the culture medium. Thus, a certain concentration of *D. leptopodum* alkaloids can change the cell permeability of *K. pneumoniae* and has a significant antibacterial effect on *K. pneumoniae* [87].

Currently, the research on the cell membrane-damaging mechanism of alkaloids is mainly focused on finding more membrane-localized targets, which may be involved in very different processes, such as proton motive force (PMF), electron flow, nutrient uptake, or other unrelated enzyme activities [88]. In a study on isoquinoline alkaloids using a drug–target interaction network for *Macleaya cordata*, 23 targets of dihydrochelerythrine and multiple targets of other compounds were successfully fitted using reverse pharmacophore database screening technology. The improvement in the target screening efficiency provides a highly focused approach for studying antibacterial mechanisms [89].

### 3.3. Inhibition of Efflux Pumps

Drug efflux pumps are bacterial transmembrane protein complexes [90]. If the efflux pump system achieves the timely exclusion of membrane-permeable antibacterial agents, the pathogenic bacterial cell avoids contact with the antibacterial agent in the membrane, which reduces the bactericidal effect of the agent and enhances the resistance of the pathogen via the membrane [91]. A large number of studies have shown that *E. coli* biofilms have a higher drug resistance than planktonic cells, which is associated with an increased expression of efflux pump genes in biofilms [92], which may lead to an increase in the distribution density or activity of cell membrane efflux pumps. While this process can lead to drug resistance of the biofilm-forming strains, the inhibition of efflux pump protein expression can extend the contact time between antibacterial agents and pathogenic bacteria in the membrane, which may eventually achieve sterilization.

The antibacterial effect of *Callistemon citrinus* alkaloids was determined by an antibacterial sensitivity test for the MIC and minimum bactericidal concentration (MBC) against *S. aureus* and *P. aeruginosa,* which were cultured using the broth culture method. The alkaloid extracts from *C. citrinus* and *Vernonia adoensis* had strong antibacterial properties with MICs of 0.025 and 0.21 mg/mL against *S. aureus* and *P. aeruginosa,* respectively. The MBC of the *C. citrinus* extract was 0.835 mg/mL against *S. aureus*. Rhodamine 6G fluorescent dye was used to determine the effect of the extract on drug accumulation. This fluorescent dye is actively pumped out by two ATP-dependent efflux pumps in bacteria [93]. The accumulation of rhodamine 6G due to *C. citrinus* alkaloids was the highest, with an increase of 121% compared with that in the glucose control. *S. aureus* was less susceptible to external pump inhibitors, and the cumulative amount increased by 114%. *P. aeruginosa* was more susceptible to external pump inhibition than *S. aureus*. Furthermore, jatrorrhizine interacted with NorA via hydrogen bonds and electrostatic interactions, which significantly inhibited the bacterial drug efflux and NorA expression at the mRNA level [94]. *P. aeruginosa* was the most susceptible target organism to efflux pump inhibition by alkaloids extracted from *C. citrinus* [82]. In another study, the researchers evaluated the efflux inhibitory activity of 13 alkaloid compounds as potential efflux pump inhibitors (EPI) against MRSA. Quinine isolated from the *Cinchona* tree bark, piperine isolated from *Piperaceae*, and harmaline isolated from *Peganum harmala* reduced the ethidium bromide (EtBr) MIC by approximately two-fold [95]. This result indicated a synergistic effect. However, the energy sources contributing to the synergy should be studied further.

In addition, in vitro studies demonstrated that inhibitors of multidrug-resistance (MDR) efflux pumps also have synergistic effects with other antibacterial plant compounds [96]. Thus, the inhibition of the expression of efflux pump genes prevents the depletion of intracellular antibacterial levels by the activity of the MDR efflux pumps, which prevents bacterial resistance and improves the antibacterial effects [82]. Rational development and utilization of efflux pump inhibitors may promote the recycling of existing antibiotics [97].

### 3.4. Inhibition of Bacterial Metabolism

Alkaloids may also exert antibacterial activities by interfering with the primary and energy metabolisms in bacteria to block bacterial toxins or inhibit bacterial growth. Adenosine triphosphate (ATP) is among the potential targets. It is usually synthesized via respiration, representing the most direct energy source in organisms that ensures the energy supply for various life activities in cells. ATP not only plays an important role in respiration and primary metabolism but it also acts as the energy source for some enzyme reactions. Therefore, the inhibition of ATP synthase affects many normal metabolic processes in microorganisms, which can lead to biological death [98].

Berberine can affect the carbohydrate metabolism of *Streptococcus pyogenes* (Group A streptococcus, GAS) by increasing the conversion and uptake of carbohydrates, as well as reducing carbohydrate consumption. Berberine increased the conversion and absorption of carbohydrates by stimulating the conversion pathway of other substances to monosaccharides and their derivatives. In addition, the ATP-binding cassette transporter and phosphotransferase systems involved in carbohydrate uptake were also upregulated by berberine. To reduce the consumption of carbohydrates, the pathways involved in glycolysis, purine metabolism, pyrimidine metabolism, fructose and mannose metabolism, and fatty acid biosynthesis were suppressed in the berberine treatment groups. Due to the disturbance of the carbohydrate metabolism, the redox and energy-substance metabolisms become unbalanced. These results indicated the stimulation of excessive ROS by berberine in GAS [99], which ultimately inhibited the bacteria. Alkaloids from *D. leptopodum* (Maxim) Fedde can inhibit *K. pneumoniae,* likely by infiltrating the cells, inhibiting the activity of intracellular enzymes, and disturbing the normal metabolic activities of cells, all of which contribute to the inhibition of the bacteria [87]. *Aconitum* biological alkaline solution and *Dendrobium* biological alkaline solution also displayed an inhibitory effect on the metabolism of *S. aureus* [100].

Some individual alkaloids have dual effects on bacterial metabolism. For instance, the *Aconitum carmichaeli* alkaloids have the opposite effect on the metabolism of *E. coli* and *S. aureus*. The *A. carmichaeli* alkaloids have a bacteria-promoting effect on *E. coli*, whereas the effect on the metabolism of *S. aureus* is antibacterial [101]. Hence, it is necessary to select suitable alkaloids for different bacteria.

### 3.5. Other Mechanisms

**Figure 1:**
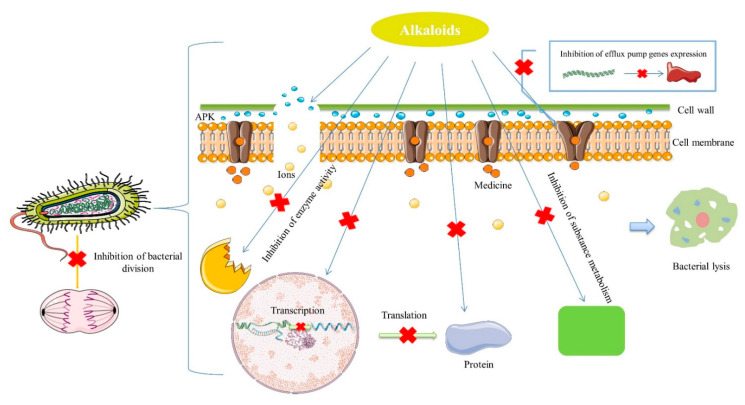
Antibacterial mechanism of natural alkaloids.

In addition to the well-established antibacterial mechanisms, alkaloids can also inhibit the activity of bacterial functional proteases and affect DNA topoisomerase and respiration. The compounds have a protective effect on the intestinal mucosa and can interact with the intestinal flora. DNA topoisomerase is an enzyme that regulates the DNA superhelical state and exists in the nucleus. It can catalyze the cleavage and binding of DNA strands, thereby affecting the topological state of DNA [102]. For example, camptothecin captures and cleaves the intermediate complex of DNA topoisomerase I [103]. A gene chip analysis revealed that berberine could inhibit the glutamine synthetase in *S. aureus*, depleting the level of glutamine as an important amino acid in bacteria [104], which then inhibits the bacteria. Berberine can be used as an antagonist of bacterial lipopolysaccharide (LPS). It inhibits the LPS/TLR4 signaling pathway, blocks the secretion of critical inflammatory factors, including nuclear factor-*κ*B (NF-*κ*B), interleukin (IL)-6, tumor necrosis factor (TNF)-*α*, and interferon (IFN)-*β*, protects against gastrointestinal mucosa damage caused by LPS, and functions as an antibacterial agent [105]. Moreover, it can upregulate the expression of caspase-1p 10 and IL-1*β* in macrophages that phagocytize *E. coli*, which activates the AMPK pathway [106] and initiates AMPK-mediated autophagy of macrophages [107], which produces an antibacterial effect. Berberine can reduce the production of ATP and NADH in intestinal bacteria, which may also be one of the mechanisms of bacterial inhibition [108]. Highly polar imine groups have been found to react with NADH or non-target nucleophilic substances in biological fluids to form stable neutral phenanthridine derivatives [109]. Thus, isoquinoline alkaloids may affect bacterial respiration. Figure 1 shows the antibacterial mechanism of natural alkaloids.

## 4. Conclusions

Among natural products, it is generally accepted that phytochemicals are less potent anti-infectives than agents of microbial origin, i.e., antibiotics [110]. However, with the increase in bacterial resistance, there are multidrug-resistant bacteria, such as *S. aureus*, which became resistant to penicillin, amoxicillin, methicillin, oxacillin, and other antibiotics, by obtaining mobile gene elements encoding the resistance factors via horizontal gene transfer [111]. Facing an imminent antibiotic resistance crisis, we should vigorously screen for new antibiotics or find alternative intervention strategies to eliminate drug resistance [112].

Alkaloids have the characteristics of broad-spectrum antibiotics but with fewer adverse reactions and a low tendency to drug resistance. The gradually achieved maturity of the alkaloid extraction technology is also the main reason for the further development of these compounds. For example, multi-component analysis (MCA) is used for microwave-assisted extraction of alkaloids [113]. In addition, new technologies, such as high-throughput screening and computational methods, can be exploited to improve the extraction rate of alkaloids. The antibacterial activity of alkaloids is related to many factors, such as the content of active substances in the extract, which are affected by internal and external factors. Internal factors are affected by plant genetics, whereas external factors are more affected by plant physiology and ecology [114]. The bioavailability of alkaloids is also a widely shared concern. Generally, alkaloids can form salts with acids and dissolve in water. Their bases are lipophilic, which facilitates absorption from the gastrointestinal tract by lipophilic diffusion [115]. The average bioavailability of different alkaloids ranges from 0.27% to 64.6%. The data are from the Traditional Chinese Medicine Systems Pharmacology Database and Analysis Platform (TCMSP; https://tcmspw.com/tcmsp.php, accessed on 12 March 2021). However, recent research showed that formulations composed of alkaloids, phospholipids, hydrophobic components, and surfactants spontaneously formed microemulsions and sub-microemulsions in vivo, which improved the oral absorption of alkaloids with low bioavailability [116]. Therefore, several aspects should be considered in the development of clinical antibacterial drugs based on the characteristics and benefits of the antibacterial activity of alkaloids. Firstly, the antibacterial mechanism of some alkaloids has not been fully elucidated, and there are few studies on drugs that inhibit bacterial metabolism. In addition, most of the current studies were carried out in vitro and relatively few were carried out in vivo. Future clinical research should use modern scientific methods to provide important discoveries and innovations in antibacterial drugs. Second, in future research, we should focus on the relationship between the antibacterial activity of alkaloids and their structures, followed by optimization of the alkaloids through structural changes. As the lead compounds in the development of botanical antibacterial drugs and/or their ancillary compounds, we may develop an antibacterial agent that can be widely used in clinical practice. Third, due to the increase in bacterial resistance, the combination of natural adjuvants, such as efflux pump inhibitors, with new and old antibiotics is still a new approach to combat antibiotic resistance [82]. The application of modern technology to explore the mechanism of drug combinations can lead to unexpected results. Fourth, over an extended initial period, the development of new alkaloid-based drugs will be affected by the identification process because of the low alkaloid content in the natural source materials and the structural complexity of some compounds. Moreover, alkaloids are stereoselectively synthesized natural products, and it is very difficult and expensive to recapitulate their synthesis in the laboratory. Interestingly, some alkaloids can be recovered by industrial “waste utilization” extraction using non-medicinal plant parts, such as the stems and leaves of *Sophora flavescens* and *Sophora alopecuroides*, which contain antibacterial alkaloids [117,118]. Finally, the development of new antibacterial drugs should consider the characteristics of multi-factor, multi-site, multi-link, and multi-mechanism of bacterial infections, and they should be optimized to achieve an appropriate dose at an acceptable level of toxicity. Therefore, a full understanding of the interaction between structurally complex drugs and the host and the identification of new potential targets are key to the study of related mechanisms.

In conclusion, research on the antibacterial activity of alkaloids has important practical significance for the research and development of new plant-derived antibacterial agents. Currently, it is still necessary to extensively perform basic and clinical research to identify and verify new drug targets and new drugs. Alkaloids are secondary metabolites and renewable resources in plants. An in-depth analysis of the antibacterial effects and mechanisms of alkaloids is expected to inspire and promote the research and development of new antibacterial drugs with high efficiency, broad-spectrum antibacterial activity, low toxicity, and a diminished tendency to induce drug resistance.
